# JAVEMACS-D: claims database analysis of avelumab maintenance therapy for advanced urothelial carcinoma in Japan

**DOI:** 10.1038/s41598-025-25515-1

**Published:** 2025-11-25

**Authors:** Takashi Kobayashi, Hiroshi Kitamura, Yuka Furukawa, Michihiro Shono, Takayuki Ito, Eiji Kikuchi

**Affiliations:** 1 Department of Urology, Kyoto University Graduate School of Medicine, Kyoto, Japan; 2 Department of Urology, Faculty of Medicine, University of Toyama, Toyama, Japan; 3 Clinical Research Department, Ark Medical Solutions Inc., Tokyo, Japan; 4 Medical Department, Merck Biopharma Co., Ltd., Tokyo, Japan, an affiliate of Merck KGaA, Tokyo, Japan; 5 Department of Urology, St Marianna University School of Medicine, Kanagawa, Japan

**Keywords:** Avelumab maintenance, Claims database, Platinum-based chemotherapy, Real world, Subsequent treatment, Time to treatment failure, Cancer, Oncology, Urology

## Abstract

JAVEMACS-D was an observational, retrospective study describing real-world characteristics, treatment patterns, and outcomes in patients with advanced urothelial carcinoma (aUC) treated with avelumab maintenance after first-line platinum-based chemotherapy (PBC) in Japan using a large claims database (Medical Data Vision). Between February 2021 and April 2023, 773 patients received avelumab maintenance; median age was 74 years, primary tumor site was bladder in 59.9% and renal pelvis/ureter in 42.7%, and first-line PBC was cisplatin-gemcitabine in 61.3% and carboplatin-gemcitabine in 36.4%. Avelumab maintenance started in 2021 in 289 patients (37.4%) and in 2022 or later in 484 (62.6%). At data cutoff (October 2023), median follow-up was 19.3 months, and 22.0% of patients were still receiving avelumab. Among 394 patients (51.0%) who received second-line treatment, the most common was enfortumab vedotin (EV) in 47.0%. Median time to treatment failure (TTF; time from start of avelumab maintenance to discontinuation) was 4.5 months (95% CI 3.8–5.2). Median TTF2 (time from start of avelumab to discontinuation of subsequent treatment) was 10.1 months (95% CI 8.7–11.0). JAVEMACS-D represents the largest real-world dataset of avelumab maintenance therapy for aUC reported to date. Data provide insights about patient characteristics, treatment patterns, and outcomes within an evolving treatment landscape.

**Supplementary Information:**

The online version contains supplementary material available at 10.1038/s41598-025-25515-1.

## Introduction

Bladder cancer is the ninth most common site of cancer worldwide and the second most common cancer site within the urinary system^1^. In 2022, bladder cancer accounted for over 613,000 new cases and more than 220,000 deaths worldwide. Urothelial carcinoma (UC) is the most common histological type of bladder cancer, accounting for 90% of cases globally (lower tract); UC can also arise in the upper urinary tract (ureter and renal pelvis) and urethra^2,3^.

Traditionally, advanced UC (aUC) has been associated with a poor prognosis^4^. However, in recent years, significant progress has been made in developing novel therapies to improve outcomes for these patients. International treatment guidelines recommend first-line (1L) platinum-based chemotherapy (PBC) followed by avelumab maintenance therapy for patients without disease progression; this remains an important and relevant treatment option for cisplatin-eligible and -ineligible patients with aUC worldwide^2,5–7^. Other recommended 1L treatment options include enfortumab vedotin (EV) + pembrolizumab (with varying availability across countries) and nivolumab + cisplatin/gemcitabine. Monotherapy with immune checkpoint inhibitors such as pembrolizumab and atezolizumab are also approved in some countries for platinum-ineligible or cisplatin-ineligible patients with programmed death ligand 1 (PD-L1) positive tumors. Subsequent treatment options depend on the 1L regimen received and may include EV monotherapy (patients with prior PBC and/or anti–PD-[L]1 treatment), pembrolizumab monotherapy (patients with prior chemotherapy), and erdafitinib monotherapy (patients with *FGFR3* genetic alterations).

In Japan, avelumab was approved in February 2021 as maintenance therapy following chemotherapy for patients with curatively unresectable UC^8^. Recently, EV + pembrolizumab as well as nivolumab + cisplatin/gemcitabine were approved as 1L therapies in Japan in December 2024^9,10^. Available options for later-line treatment in Japan include pembrolizumab monotherapy (approved in January 2018)^11^ and EV monotherapy (approved in September 2021)^12^.

The approval of avelumab maintenance therapy worldwide was based on results from the JAVELIN Bladder 100 phase 3 trial, which demonstrated that in patients with aUC without disease progression after 1L PBC, avelumab + best supportive care (BSC) significantly prolonged overall survival (OS) and progression-free survival (PFS) vs BSC alone and had an acceptable safety and tolerability profile^13,14^. In long-term analyses (≥ 2 years of follow-up in all patients), the median OS from randomization with avelumab + BSC vs BSC alone was 23.8 vs 15.0 months (hazard ratio [HR], 0.76 [95% CI, 0.63–0.91]; p = 0.0036), and the median PFS was 5.5 vs 2.1 months (HR, 0.54 [95% CI, 0.46–0.64]; p < 0.0001), respectively. In a post hoc subgroup analysis of patients enrolled in Japan that included 36 patients treated with avelumab maintenance, efficacy and safety findings were generally consistent with those reported in the overall population (avelumab + BSC vs BSC alone: median OS, 24.7 vs 18.7 months [HR, 0.81; 95% CI, 0.41–1.58]; median PFS, 5.6 vs 1.9 months [HR, 0.63; 95% CI, 0.36–1.11])^15^.

Subsequently, post-marketing surveillance was performed in Japan, which confirmed the real-world safety and effectiveness in a large population of patients who started avelumab maintenance in 2021 (N = 453)^16^. Other real-world studies in various countries have also demonstrated the effectiveness and safety of avelumab maintenance in clinical practice, including a small study in Japan^17–23^. Some studies have examined treatments administered after avelumab 1L maintenance in routine clinical practice^17,21,24,25^. However, additional studies in large populations are needed to obtain further insights about real-world patient characteristics, treatment patterns, and outcomes in patients who have received avelumab maintenance.

Here, we report results from JAVEMACS-D, a descriptive real-world study of patients with aUC treated with avelumab maintenance using one of the largest claims databases in Japan (Medical Data Vision [MDV]), which covered a time period of > 2.5 years after avelumab was first approved.

## Results

### Baseline characteristics

**Fig. 1:**
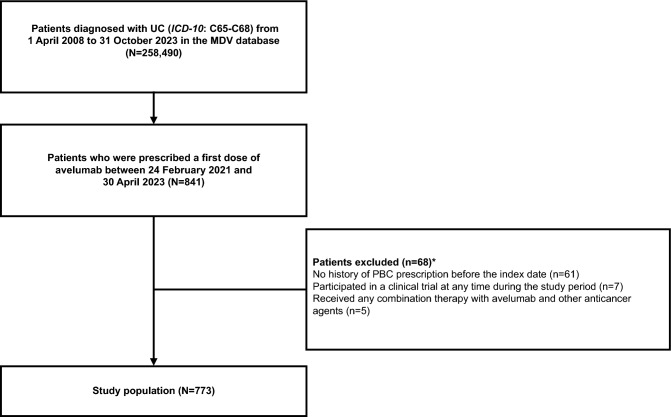
Patient flow chart. *ICD-10,* International Classification of Diseases, Tenth Revision; *MDV,* Medical Data Vision; *PBC,* platinum-based chemotherapy; *UC,* urothelial carcinoma. *Some patients met > 1 criteria for exclusion.

By data cutoff (31 October 2023), 258,490 patients diagnosed with UC were identified in the MDV database; of these, 841 patients had received a first dose of avelumab maintenance between 24 February 2021 and 30 April 2023, and 773 patients met all eligibility criteria (see “Patients and Methods: Study design and patient population” section) and were included in the study population (Fig. 1). At the index date (start of avelumab maintenance treatment), median age was 74 years (range, 40–98 years), and 189 patients (24.5%) were aged ≥ 80 years (Table 1). Avelumab maintenance therapy started in 2021 in 289 patients (37.4%) and in 2022 or later in 484 (62.6%); patient characteristics were generally similar across these subgroups. Baseline characteristics in subgroups defined by second-line (2L) treatment are shown in Supplementary Table S1.

**Table 1:** Baseline characteristics in the overall population and in subgroups defined by avelumab start date.

	Overall population (N = 773)	Start of avelumab maintenance	
		2021 (n = 289)	2022 or later (n = 484)
**Age**			
Median (IQR) [range], years	74 (69–79) [40–98]	74 (69–79) [44–89]	74 (69–80) [40–98]
< 65 years, n (%)	106 (13.7)	35 (12.1)	71 (14.7)
65 to < 75 years, n (%)	309 (40.0)	127 (43.9)	182 (37.6)
75 to < 80 years, n (%)	169 (21.9)	69 (23.9)	100 (20.7)
≥ 80 years, n (%)	189 (24.5)	58 (20.1)	131 (27.1)
**Sex, n (%)**			
Male	576 (74.5)	219 (75.8)	357 (73.8)
Female	197 (25.5)	70 (24.2)	127 (26.2)
**Body mass index**			
Median (IQR), kg/m^2^	22.9 (20.9–25.0)	23.2 (21.3–25.2)	22.7 (20.7–24.8)
< 18.5 kg/m^2^, n (%)	58 (7.5)	20 (6.9)	38 (7.9)
18.5 to < 25 kg/m^2^, n (%)	508 (65.7)	184 (63.7)	324 (66.9)
≥ 25 kg/m^2^, n (%)	189 (24.5)	77 (26.6)	112 (23.1)
Unknown	18 (2.3)	8 (2.8)	10 (2.1)
**Smoking status, n (%)**			
Yes	350 (45.3)	138 (47.8)	212 (43.8)
No	307 (39.7)	105 (36.3)	202 (41.7)
Unknown	116 (15.0)	46 (15.9)	70 (14.5)
**Primary tumor site, n (%)**			
Bladder	463 (59.9)	178 (61.6)	285 (58.9)
Renal pelvis and ureter	330 (42.7)	121 (41.9)	209 (43.2)
Urethra	6 (0.8)	1 (0.3)	5 (1.0)
**Metastatic site, n (%)**			
Lung	148 (19.1)	56 (19.4)	92 (19.0)
Liver	48 (6.2)	24 (8.3)	24 (5.0)
Bone	102 (13.2)	45 (15.6)	57 (11.8)
Peritoneum	19 (2.5)	8 (2.8)	11 (2.3)
Other	39 (5.0)	17 (5.9)	22 (4.5)
**Hospital scale, n (%)**			
< 200 beds	11 (1.4)	3 (1.0)	8 (1.7)
200 to < 500 beds	351 (45.4)	113 (39.1)	238 (49.2)
≥ 500 beds	411 (53.2)	173 (59.9)	238 (49.2)
**Designated cancer care hospital, n (%)**			
Yes	692 (89.5)	265 (91.7)	427 (88.2)
No	81 (10.5)	24 (8.3)	57 (11.8)

*IQR,* interquartile range.

### 1L PBC treatment

The 1L PBC regimen before avelumab maintenance was cisplatin + gemcitabine in 474 patients (61.3%); carboplatin + gemcitabine in 281 (36.4%); dose-dense methotrexate, vinblastine, doxorubicin, and cisplatin (ddMVAC) in 8 (1.0%); and other PBC regimens in 10 (1.3%) (Table 2). The number of PBC cycles received was 1–3 in 139 (18.0%), 4 in 260 (33.6%), 5–6 in 224 (29.0%), and ≥ 7 in 150 (19.4%), and the median dosing period from the start date of 1L PBC to the date of first dose of avelumab was 22.3 weeks (IQR, 17.0–33.7). Patients who started avelumab maintenance in 2022 or later received fewer cycles of PBC than those who started treatment in 2021 (median 4 vs 5; 4 cycles in 36.2% vs 29.4%, ≥ 7 cycles in 16.5% vs 24.2%, respectively). In the overall population, the platinum dose was reduced in 195 patients (25.2%). The median treatment-free interval between the last dose of PBC and first dose of avelumab was 5.0 weeks (IQR, 3.6–6.9); the treatment-free interval was < 4 weeks in 239 patients (30.9%), 4–10 weeks in 474 (61.3%), and > 10 weeks in 60 (7.8%). Characteristics of 1L PBC in subgroups defined by 2L treatment are shown in Supplementary Table S2.

**Table 2:** Characteristics of 1L PBC prior to avelumab maintenance in the overall population and in subgroups defined by avelumab start date.

	Overall population (N = 773)	Start of avelumab maintenance	
		2021 (n = 289)	2022 or later (n = 484)
**Regimen, n (%)**			
Cisplatin + gemcitabine	474 (61.3)	178 (61.6)	296 (61.2)
Carboplatin + gemcitabine	281 (36.4)	104 (36.0)	177 (36.6)
ddMVAC	8 (1.0)	1 (0.3)	7 (1.4)
Other*	10 (1.3)	6 (2.1)	4 (0.8)
**Cycles**			
Median (IQR)	4 (4–6)	5 (4–6)	4 (4–6)
1–3 cycles, n (%)	139 (18.0)	43 (14.9)	96 (19.8)
4 cycles, n (%)	260 (33.6)	85 (29.4)	175 (36.2)
5–6 cycles, n (%)	224 (29.0)	91 (31.5)	133 (27.5)
≥ 7 cycles, n (%)	150 (19.4)	70 (24.2)	80 (16.5)
**Dose reduction, n (%)**	195 (25.2)	68 (23.5)	127 (26.2)
First cycle when dose reduction occurred, median (IQR)	3 (2–4)	3 (2–4)	3 (2–4)
**Changed platinum agent, n (%)**	69 (8.9)	29 (10.0)	40 (8.3)
First cycle when platinum agent was changed, median (IQR)	3 (3–5)	3 (3–5)	3 (3–5)
**Dosing period, median (IQR), weeks**^†^	22.3 (17.0–33.7)	24.3 (17.1–39.0)	21.9 (17.0–30.9)
**Treatment-free interval, median (IQR), weeks**^‡^	5.0 (3.6–6.9)	5.0 (3.4–6.7)	5.0 (3.6–7.0)
< 4 weeks, n (%)	239 (30.9)	89 (30.8)	150 (31.0)
4–10 weeks, n (%)	474 (61.3)	178 (61.6)	296 (61.2)
> 10 weeks, n (%)	60 (7.8)	22 (7.6)	38 (7.9)

*1L,* first line; *ddMVAC,* dose-dense methotrexate, vinblastine, doxorubicin, cisplatin; *IQR,* interquartile range; *PBC,* platinum-based chemotherapy.

*Treatment with a cisplatin- or carboplatin-containing regimen other than cisplatin + gemcitabine, carboplatin + gemcitabine, or ddMVAC.

^†^Time from the first dose of 1L PBC to the first dose of avelumab maintenance treatment.

^‡^Time from the last dose of 1L PBC to the first dose of avelumab maintenance treatment.

### Avelumab maintenance therapy and subsequent treatment

The median duration of avelumab maintenance was 19.1 weeks (IQR, 9.1–41.1), and the median number of cycles received was 9 (IQR, 5–18). The interval between avelumab doses showed no trend over time (Supplementary Fig. S1). In a simple linear regression of dose interval (weeks) by treatment week, the intercept was 2.34 weeks (95% CI, 2.31–2.38) and the slope was 0.002 weeks per week (95% CI, 0.001–0.003), with low explanatory power (R^2^ = 0.001).

**Fig. 2:**
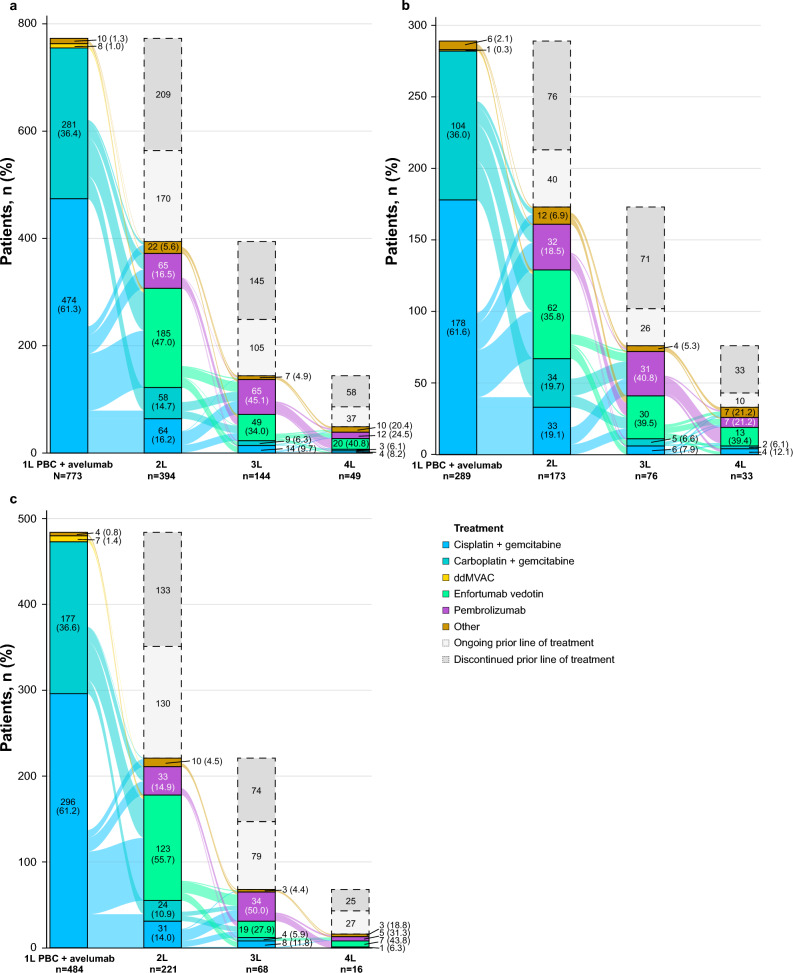
Treatment patterns. (**a**) Overall population. (**b**) Patients who started avelumab maintenance in 2021. (**c**) Patients who started avelumab maintenance in 2022 or later. *1L,* first line; *2L,* second line; *3L,* third line; *4L,* fourth line; *ddMVAC,* dose-dense methotrexate, vinblastine, doxorubicin, and cisplatin; *PBC,* platinum-based chemotherapy.

At data cutoff, 170 patients (22.0%) were still receiving avelumab maintenance, 394 (51.0%) had discontinued avelumab and received 2L treatment, and 209 (27.0%) had discontinued avelumab without 2L treatment (Fig. 2a). The most common 2L treatments were EV in 185 patients (47.0%), pembrolizumab in 65 (16.5%), cisplatin + gemcitabine in 64 (16.2%), and carboplatin + gemcitabine in 58 (14.7%). Third-line (3L) and fourth-line (4L) treatments were received by 144 (18.6%) and 49 (6.3%) patients of the overall population, respectively (Fig. 2a and Supplementary Table S3). The most common 3L and 4L treatments were pembrolizumab (45.1% and 24.5%, respectively) and EV (34.0% and 40.8%, respectively). Compared with patients who started avelumab maintenance in 2021, the subgroup who started treatment in 2022 or later included a higher proportion of patients who received 2L EV (55.7% vs 35.8%, respectively) and a lower proportion who received 2L PBC (cisplatin + gemcitabine in 14.0% vs 19.1%; carboplatin + gemcitabine in 10.9% vs 19.7%, respectively; Fig. 2b,c).

### Outcomes

**Fig. 3:**
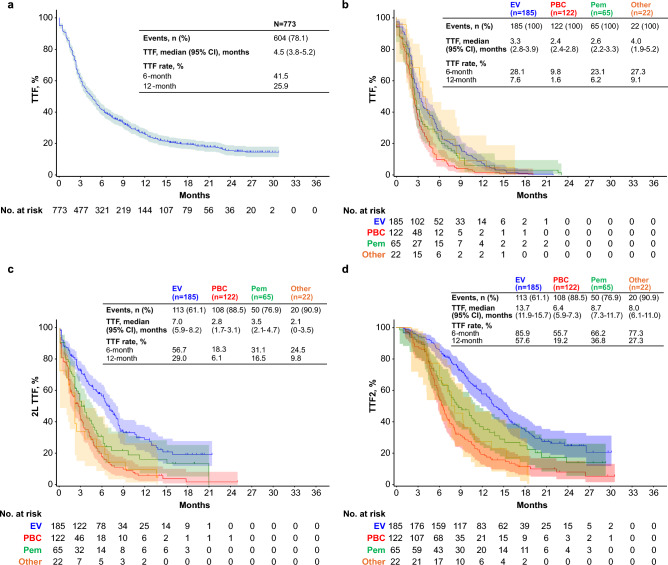
Outcomes in patients treated with avelumab maintenance. (**a**) TTF from the start of avelumab maintenance in the overall population. (**b**) TTF from avelumab maintenance by type of 2L treatment. (**c**) 2L TTF from the start of 2L treatment by type of 2L treatment. (**d**) TTF2 from avelumab maintenance by type of 2L treatment. *2L,* second line; *EV,* enfortumab vedotin; *PBC,* platinum-based chemotherapy,;*Pem,* pembrolizumab; *TTF,* time to treatment failure.

The median duration of follow-up from the start of avelumab maintenance was 19.3 months (IQR, 12.1–25.3) and from the start of 2L treatment was 14.2 months (IQR, 8.1–20.5). In the overall population, the median time to treatment failure (TTF; defined as time from the start of avelumab maintenance therapy to discontinuation for any reason) was 4.5 months (95% CI 3.8–5.2) (Fig. 3a). TTF from the start of avelumab maintenance in subgroups is shown in Supplementary Fig. S2. In the 394 patients who had discontinued avelumab and received 2L treatment, the median TTF from the start of avelumab maintenance therapy was generally consistent among 2L treatment groups (Fig. 3b). The median 2L TTF (defined as time from the start of 2L treatment to discontinuation for any reason) was 4.0 months (95% CI 3.3–4.7) overall and in patients who received 2L EV, PBC, pembrolizumab, or other treatments it was 7.0 months (95% CI 5.9–8.2), 2.8 months (95% CI 1.7–3.1), 3.5 months (95% CI 2.1–4.7), and 2.1 months (95% CI 0.0–3.5), respectively (Fig. 3c). The median TTF2 (defined as time from start of avelumab maintenance therapy to discontinuation of subsequent treatment for any reason) was 10.1 months (95% CI 8.7–11.0) overall and in patients who received 2L EV, PBC, pembrolizumab, or other treatments it was 13.7 months (95% CI 11.9–15.7), 6.4 months (95% CI 5.9–7.3), 8.7 months (95% CI 7.3–11.7), and 8.0 months (95% CI 6.1–11.0), respectively (Fig. 3d). Median OS (defined as time from start of avelumab maintenance therapy to the date of death from any cause) was not estimable (NE) (95% CI, NE-NE), and 12- and 24-month OS rates were 87.7% and 80.5%, respectively.

## Discussion

JAVEMACS-D is a descriptive study that represents the largest real-world dataset of patients with aUC treated with avelumab maintenance therapy in clinical practice reported to date. Patient characteristics in the study population differed from those in the avelumab + BSC arm of JAVELIN Bladder 100^13,26^, including a higher age (median, 74 vs 68 years; ≥ 75 years in 46.3% vs 24.3% and ≥ 80 years in 24.5% vs 8.0%) and a higher proportion of patients with upper urinary tract primary tumors (42.7% vs 30.3%), respectively, consistent with other real-world studies in Japan^16,23,27^. The 1L PBC administered was cisplatin + gemcitabine in 61.3% and carboplatin + gemcitabine in 36.4%, indicating higher cisplatin use than in JAVELIN Bladder 100 (52.3%)^13^, which is also consistent with previous studies in Japan^16,23,27^.

The JAVEMACS-D population included some patients whose 1L PBC characteristics differed from the eligibility requirements of JAVELIN Bladder 100 (4–6 cycles of gemcitabine + cisplatin or carboplatin followed by a treatment-free interval of 4–10 weeks)^13^. In particular, 18.0% and 19.4% had received 1–3 or ≥ 7 cycles of PBC, respectively, and the treatment-free interval was < 4 or > 10 weeks in 30.9% and 7.8%, respectively, illustrating the heterogeneity of the JAVEMACS-D study population. Within the study population, the subgroup who received ≥ 7 cycles of PBC may have included patients who started PBC prior to the approval of avelumab maintenance, and who switched treatment once it became available. In treatment guidelines, recommendations for the number of cycles of PBC prior to avelumab maintenance are aligned to the study design of JAVELIN Bladder 100^2,5–7,13^; in clinical practice, physicians may switch to avelumab maintenance as appropriate based on outcomes with 1L PBC and individual patient and disease characteristics. In this study, the interval between avelumab cycles was approximately 2 weeks in most patients, consistent with prescribing information^8,28^, which supports the feasibility and tolerability of treatment in clinical practice.

Patient characteristics were generally similar in subgroups who started avelumab therapy in 2021 vs 2022 or later. However, the number of prior PBC cycles administered was lower in the 2022 or later subgroup vs the 2021 subgroup, suggesting an increasing preference to switch from 1L PBC to avelumab maintenance earlier in treatment, potentially to avoid additional PBC cycles once initial treatment benefits have been obtained^29^. The median TTF from start of avelumab maintenance was 4.5 months, with no major differences observed in most subgroups, including older age subgroups (e.g., ≥ 75 or ≥ 80 years). This finding is informative given that patients with aUC tend to be older and suggests that avelumab maintenance results in generally similar outcomes across age subgroups. However, causal or comparative effectiveness inferences cannot be drawn because of the observational design of the study. The median TTF in this study was similar to that of two other real-world studies in Japan, which reported a median TTF of 4.6 months^16,23^. TTF has been evaluated in real-world studies in other cancer types^30,31^ and provides an indication of clinical benefit that is distinct from progression-free survival, which could not be analyzed in this population. In clinical practice, treatment may be discontinued for reasons other than disease progression (eg, side effects, decreased patient quality of life, patient preference), which are all captured in the TTF analysis; thus, TTF provides an overall evaluation that combines effectiveness and tolerability^32^.

Median OS from the start of avelumab maintenance in this population was NE. However, this analysis should be interpreted with consideration of the characteristics of the MDV claims database, which reliably captures inpatient deaths but underestimates out-of-hospital deaths, records events typically at month-level, and has partial national hospital coverage leading to loss of follow-up upon patient transfer^33^. Other studies using data from the MDV database have reported OS in different settings^34–36^; however, the authors cautioned that deaths after hospital discharge may not be recorded. Our anchoring at the start of avelumab maintenance (earlier in the treatment journey), coupled with ongoing therapy at data cutoff and underestimation of out‑of‑hospital death, may have reduced events within follow‑up and contributed to median OS being NE.

At data cutoff, 170 patients (22.0%) were still receiving avelumab maintenance therapy. Of 603 patients who discontinued avelumab, 394 (65.3%) received 2L therapy and 209 (34.7%) did not receive any subsequent treatment. Among the 394 patients who received 2L treatment, EV was the most common subsequent treatment (47.0% of 2L, 34.0% of 3L, and 40.8% of 4L patients). Use of EV as 2L treatment increased over time (55.7% vs 35.8% of 2L patients who started avelumab in 2022 or later vs 2021, respectively), consistent with dates of regulatory approval (September 2021) and launch (November 2021) of EV monotherapy in Japan for patients with unresectable UC following disease progression after prior chemotherapy^12,37^. The relatively high transition rate to 2L treatment after discontinuation of avelumab may be due to Japan having a universal health insurance system, which provides access to recently approved therapies, such as EV. In real-world studies of avelumab 1L maintenance in the US and France, 70% to 73% of patients who discontinued avelumab received 2L treatment^17,21^. Overall, these data indicate that most patients who discontinue avelumab can receive additional treatments. Patients who did not receive 2L treatment in this study may include those who had disease stabilization with avelumab maintenance or who were unable to receive further treatment because of general health deterioration following disease progression. Of note, patients who did not receive 2L treatment were generally older than those who receive 2L treatment.

Baseline characteristics were generally consistent across subgroups defined by type of 2L treatment administered. However, patients who received 2L EV had received more avelumab cycles than those who received 2L PBC or pembrolizumab. In subgroup analyses of patients who received different 2L treatments, 2L TTF and TTF2 were longer in patients who received 2L EV than in patients who received 2L PBC, pembrolizumab, or other treatments, suggesting that EV may be an effective treatment option in patients who have received 1L PBC and avelumab maintenance. Outcomes with 2L EV after avelumab maintenance have also been reported in other real-world studies, with median OS and PFS from start of 2L EV ranging from 11.2–13.5 months and 4.9–7.9 months, respectively^21,24,25,38^; these outcomes are similar to data from the EV-301 phase 3 trial of EV vs physician’s choice of chemotherapy in patients with prior PBC and immune checkpoint inhibitor treatment (median OS, 12.91 months; median PFS, 5.55 months)^39^. To our knowledge, no previous studies have reported outcomes from start of 2L PBC after avelumab 1L maintenance. However, in a real-world study in France, the median OS from the start of avelumab 1L maintenance was 16.7 months in patients who received 2L PBC, 13.6 months in patients who received 2L treatment with nonplatinum chemotherapy, and 36.0 months in patients who received 2L EV^17^.

This study has several limitations due to the nature of the MDV database, which provided all data for the study. The MDV database only records diagnoses linked to insurance claims for procedures or medications, which may lead to underestimation of comorbidities/underlying conditions and the occurrence/grading of adverse events; therefore, detailed characterization of safety and toxicity was not performed in this claims-based study. Additionally, available data do not indicate whether adverse events were associated with drug therapy. Consequently, adverse events were not evaluated in this study. Complementary safety evidence for avelumab maintenance in Japan has been evaluated in contemporaneous post-marketing surveillance^16^. Patients who move between healthcare facilities cannot be identified because of the lack of a unique patient identification number, which means that such patients may be duplicated within the study population or may be lost to follow-up. Medical coding in MDV records is based on Japanese diagnostic codes, which are assigned by trained doctors but may be subject to human error. MDV data do not include information on drug dose, tumor response, or reason for treatment discontinuation; however, the MDV can capture information on the vial dose, number of vials, and the frequency of administration, which can be used to infer treatment characteristics such as dose reduction. As discussed earlier, the MDV database captures “inpatient” death only, meaning that death rates may be underestimated and survival analyses are generally overestimates; thus, OS analyses should be interpreted with caution. However, TTF analyses were performed using precise definitions for censoring and events (which included any recorded deaths), thereby providing an alternative outcome assessment in this population. JAVEMACS-D was a longitudinal, observational, retrospective study with no control group; therefore, it was not possible to confirm that patient outcomes observed were specifically due to avelumab treatment. Lasty, this study was descriptive in nature, thus formal statistical comparisons would not be appropriate and were not performed.

Overall, results from the large JAVEMACS-D study in Japan provide new insights about patient characteristics, treatment patterns, and outcomes in patients with aUC treated with avelumab maintenance in a contemporary real-world setting within an evolving treatment landscape. Future studies are needed to investigate whether a treatment sequence of 1L PBC, avelumab maintenance, and 2L EV can contribute to improving the prognosis of patients with aUC.

## Patients and methods

### Study design and patient population

JAVEMACS-D was a longitudinal, observational, retrospective study based on secondary data from the MDV database, which collects reimbursement data from more than 400 hospitals and covers more than 30 million patients in Japan^33^. The study population included patients aged ≥ 18 years who had a confirmed diagnosis of UC in the bladder, renal pelvis, ureter, or urethra based on *International Classification of Diseases—Tenth Revision* (*ICD-10*; codes C65-68), and who received the first dose of avelumab between 24 February 2021 and 30 April 2023 and within 180 days after the last dose of PBC (cisplatin- or carboplatin-containing regimens only). In this claims database, patients were initially identified using UC diagnostic codes and the analysis cohort of patients with aUC was operationally defined by receipt of 1L PBC followed by avelumab maintenance. Switching between platinum agents (cisplatin to carboplatin and vice versa) prior to avelumab was permitted. Patients who received any combination therapy involving avelumab and other anticancer agents, who switched from PBC to other anticancer regimens before starting avelumab, or who participated in a clinical trial in UC during the study period were excluded. For each patient, the index date was defined as the date of the first dose of avelumab, 1L chemotherapy was defined as PBC immediately prior to the first dose of avelumab, and the start of the 1L PBC was defined as the date of the first dose of 1L chemotherapy received for aUC. The data cutoff date was 31 October 2023 to ensure a minimum observation period of 6 months in all patients. The follow-up period was defined as the period from the index date to the end of the study period. The date of the first dose of an anticancer treatment received after the last dose of avelumab was defined as the start date for 2L treatment; no time limit between the date of the last dose of avelumab and the date of the next anticancer treatment was established. Dose reductions for platinum agents were defined as a dose > 15% lower than the initial dose.

All data for this study were routinely collected via the MDV database in accordance with its own policies, procedures, and statutory obligations. Health claims data were collected from all insurance types. Because this was a noninterventional study that analyzed secondary data, which were fully anonymized, informed consent was not required. Approval from the independent ethics committee/institutional review board of the nonprofit organization MINS (Tokyo, Japan) was obtained before starting the study.

### Objectives and analyses

The primary objectives of the study were to describe: (1) baseline characteristics of patients with aUC who received avelumab maintenance; (2) characteristics of 1L PBC prior to avelumab maintenance; and (3) subsequent treatments after avelumab maintenance. Subgroup analyses were performed in patients who started avelumab maintenance in 2021 vs 2022 or later. The secondary objective was to evaluate outcomes of avelumab maintenance treatment, including TTF (defined as time from the start of avelumab maintenance therapy to discontinuation for any reason, including death), TTF2 (defined as time from start of avelumab maintenance therapy to discontinuation of subsequent treatment for any reason, including death), 2L TTF (defined as time from the start of 2L treatment to discontinuation for any reason, including death), and OS (defined as time from start of avelumab maintenance therapy to the date of in-patient death due to any cause). For analyses of TTF, TTF2, and 2L TTF, the final prescription date was considered the event date if a subsequent treatment was started or if survival for ≥ 90 days from the final prescribed date was confirmed and no subsequent treatment was received. Patients who had no data regarding discontinuation within 90 days from the last observation date were censored on the last observation date. Regarding OS, only the date of death during hospitalization could be captured as an event due to the characteristics of the MDV database. If death was not recorded, data were censored at 90 days after the last visit date or the date of data cutoff, whichever occurred first.

This study was descriptive in nature; therefore, formal sample size calculations were not applicable, and all eligible patients identified were included. Continuous variables (including duration of follow-up) were summarized using descriptive statistics, and categorical variables were summarized as frequencies and percentages. A simple linear regression of avelumab dosing interval (weeks) on treatment week was fitted to assess temporal trends; the model intercept and slope were reported with 95% confidence intervals and the coefficient of determination (R^2^) was calculated. The Kaplan–Meier method was used to estimate median TTF, TTF2, 2L TTF, and OS, and corresponding 95% CIs were calculated using log transformation. Data management and analysis were performed using SAS® software version 9.4.

## Supplementary Information

Supplementary Information.

## Data Availability

Any requests for data by qualified scientific and medical researchers for legitimate research purposes will be subject to Merck’s Data Sharing Policy. All requests should be submitted in writing to Merck’s data sharing portal (https://www.merckgroup.com/en/research/our-approach-to-research-and-development/healthcare/clinical-trials/commitment-responsible-data-sharing.html). When Merck has a co-research, co-development, or co-marketing or co-promotion agreement, or when the product has been out-licensed, the responsibility for disclosure might be dependent on the agreement between parties. Under these circumstances, Merck will endeavor to gain agreement to share data in response to requests.

## Declarations

### Study registration

The study was registered on jRCT (Japan Registry of Clinical Trials). Registration number: jRCT1031240122. https://jrct.mhlw.go.jp/en-latest-detail/jRCT1031240122.
