# Supplementary material for: JAVEMACS-D: claims database analysis of avelumab maintenance therapy for advanced urothelial carcinoma in Japan
Source: Sci Rep. 2025 Nov 25;15:41773. doi: 10.1038/s41598-025-25515-1 (PMC12647644; doi:10.1038/s41598-025-25515-1)
Supplement: Supplementary file 1 — Supplementary Information. [file 41598_2025_25515_MOESM1_ESM.docx]

**Title:**

JAVEMACS-D: claims database analysis of avelumab maintenance therapy for advanced urothelial carcinoma in Japan

**Author list:**

Takashi Kobayashi,^1*^ Hiroshi Kitamura,^2^ Yuka Furukawa,^3^ Michihiro Shono,^4^ Takayuki Ito,^4^ Eiji Kikuchi^5^

**Affiliations:** ^1^ Department of Urology, Kyoto University Graduate School of Medicine, Kyoto, Japan; ^2^ Department of Urology, Faculty of Medicine, University of Toyama, Toyama, Japan; ^3^ Clinical Research Department, Ark Medical Solutions Inc., Tokyo, Japan; ^4^ Medical Department, Merck Biopharma Co., Ltd., Tokyo, Japan, an affiliate of Merck KGaA; ^5^ Department of Urology, St Marianna University School of Medicine, Kanagawa, Japan

***Corresponding author:**

Prof. Takashi Kobayashi, MD, PhD
Department of Urology, Kyoto University Graduate School of Medicine, Kyoto, Japan
selecao@kuhp.kyoto-u.ac.jp

**Table S1. Baseline characteristics in subgroups defined by 2L treatment.** Abbreviations: 2L, second line; EV, enfortumab vedotin; IQR, interquartile range; PBC, platinum-based chemotherapy.

|  | **Received 2L treatment (n=394)** | **Type of 2L treatment** | | | | **Discontinued avelumab maintenance and did not receive 2L treatment (n=209)** |
| --- | --- | --- | --- | --- | --- | --- |
|  |  | **EV (n=185)** | **PBC (n=122)** | **Pembrolizumab (n=65)** | **Other (n=22)** |  |
| **Age** |  |  |  |  |  |  |
| Median (IQR) [range], years | 73.5 (69-79) [44-89] | 73.0 (68-79) [44-89] | 74.0 (70-78) [50-86] | 73.0 (71-81)  [48-89] | 73.0 (67-79) [59-85] | 75.0 (71-81) [48-90] |
| <65 years, n (%) | 56 (14.2) | 30 (16.2) | 16 (13.1) | 9 (13.8) | 1 (4.5) | 26 (12.4) |
| 65 to <75 years, n (%) | 161 (40.9) | 73 (39.5) | 49 (40.2) | 27 (41.5) | 12 (54.5) | 69 (33.0) |
| 75 to <80 years, n (%) | 90 (22.8) | 39 (21.1) | 37 (30.3) | 10 (15.4) | 4 (18.2) | 50 (23.9) |
| ≥80 years, n (%) | 87 (22.1) | 43 (23.2) | 20 (16.4) | 19 (29.2) | 5 (22.7) | 64 (30.6) |
| **Sex, n (%)** |  |  |  |  |  |  |
| Male | 292 (74.1) | 139 (75.1) | 87 (71.3) | 49 (75.4) | 17 (77.3) | 157 (75.1) |
| Female | 102 (25.9) | 46 (24.9) | 35 (28.7) | 16 (24.6) | 5 (22.7) | 52 (24.9) |
| **Body mass index** |  |  |  |  |  |  |
| Median (IQR), kg/m^2^ | 22.7 (20.8-24.8) | 22.5 (20.7-24.6) | 23.1 (20.9-25.1) | 22.5 (20.7- 25.0) | 23.1 (21.9-24.4) | 23.3 (21.0-25.1) |
| <18.5 kg/m^2^, n (%) | 28 (7.1) | 12 (6.5) | 9 (7.4) | 5 (7.7) | 2 (9.1) | 15 (7.2) |
| ≥18.5 to <25 kg/m^2^, n (%) | 265 (67.3) | 128 (69.2) | 79 (64.8) | 43 (66.2) | 15 (68.2) | 135 (64.6) |
| ≥25 kg/m^2^, n (%) | 92 (23.4) | 40 (21.6) | 31 (25.4) | 17 (26.2) | 4 (18.2) | 54 (25.8) |
| Unknown | 9 (2.3) | 5 (2.7) | 3 (2.5) | 0 | 1 (4.5) | 5 (2.4) |
| **Smoking status, n (%)** |  |  |  |  |  |  |
| Yes | 173 (43.9) | 79 (42.7) | 54 (44.3) | 32 (49.2) | 8 (36.4) | 94 (45.0) |
| No | 164 (41.6) | 77 (41.6) | 51 (41.8) | 27 (41.5) | 9 (40.9) | 83 (39.7) |
| Unknown | 57 (14.5) | 29 (15.7) | 17 (13.9) | 6 (9.2) | 5 (22.7) | 32 (15.3) |
| **Primary tumor site, n (%)** |  |  |  |  |  |  |
| Bladder | 231 (58.6) | 108 (58.4) | 74 (60.7) | 32 (49.2) | 17 (77.3) | 117 (56.0) |
| Renal pelvis and ureter | 178 (45.2) | 82 (44.3) | 54 (44.3) | 36 (55.4) | 6 (27.3) | 94 (45.0) |
| Urethra | 2 (0.5) | 2 (1.1) | 0 | 0 | 0 | 3 (1.4) |
| **Metastatic site, n (%)** |  |  |  |  |  |  |
| Lung | 81 (20.6) | 34 (18.4) | 26 (21.3) | 13 (20.0) | 8 (36.4) | 40 (19.1) |
| Liver | 28 (7.1) | 16 (8.6) | 9 (7.4) | 1 (1.5) | 2 (9.1) | 16 (7.7) |
| Bone | 62 (15.7) | 32 (17.3) | 19 (15.6) | 8 (12.3) | 3 (13.6) | 28 (13.4) |
| Peritoneum | 10 (2.5) | 5 (2.7) | 3 (2.5) | 0 | 2 (9.1) | 4 (1.9) |
| Other | 21 (5.3) | 9 (4.9) | 8 (6.6) | 3 (4.6) | 1 (4.5) | 11 (5.3) |
| **Hospital scale, n (%)** |  |  |  |  |  |  |
| <200 beds | 5 (1.3) | 3 (1.6) | 0 | 2 (3.1) | 0 | 4 (1.9) |
| ≥200 to <500 beds | 180 (45.7) | 72 (38.9) | 63 (51.6) | 36 (55.4) | 9 (40.9) | 98 (46.9) |
| ≥500 beds | 209 (53.0) | 110 (59.5) | 59 (48.4) | 27 (41.5) | 13 (59.1) | 107 (51.2) |
| **Designated cancer care hospital, n (%)** |  |  |  |  |  |  |
| Yes | 360 (91.4) | 174 (94.1) | 111 (91.0) | 55 (84.6) | 20 (90.9) | 185 (88.5) |
| No | 34 (8.6) | 11 (5.9) | 11 (9.0) | 10 (15.4) | 2 (9.1) | 24 (11.5) |
| **Start of avelumab maintenance** |  |  |  |  |  |  |
| 2021 | 173 (43.9) | 62 (33.5) | 67 (54.9) | 32 (49.2) | 12 (54.5) | 76 (36.4) |
| 2022 or later | 221 (56.1) | 123 (66.5) | 55 (45.1) | 33 (50.8) | 10 (45.5) | 133 (63.6) |

**Table S2. Characteristics of prior 1L PBC and avelumab maintenance treatment in subgroups defined by 2L treatment.** Abbreviations: 1L, first line; 2L, second line; ddMVAC, dose-dense methotrexate, vinblastine, doxorubicin, and cisplatin; EV, enfortumab vedotin; IQR, interquartile range; PBC, platinum-based chemotherapy.

|  | **Received 2L treatment (n=394)** | **Type of 2L treatment** | | | | **Discontinued avelumab maintenance and did not receive 2L treatment (n=209)** |
| --- | --- | --- | --- | --- | --- | --- |
|  |  | **EV (n=185)** | **PBC (n=122)** | **Pembrolizumab (n=65)** | **Others (n=22)** |  |
| **Regimen, n (%)** |  |  |  |  |  |  |
| Cisplatin + gemcitabine | 236 (59.9) | 106 (57.3) | 79 (64.8) | 35 (53.8) | 16 (72.7) | 126 (60.3) |
| Carboplatin + gemcitabine | 147 (37.3) | 71 (38.4) | 43 (35.2) | 28 (43.1) | 5 (22.7) | 78 (37.3) |
| ddMVAC | 5 (1.3) | 4 (2.2) | 0 | 1 (1.5) | 0 | 1 (0.5) |
| Other* | 6 (1.5) | 4 (2.2) | 0 | 1 (1.5) | 1 (4.5) | 4 (1.9) |
| **Cycles** |  |  |  |  |  |  |
| Median (IQR) | 5.0 (4.0-6.0) | 4.0 (4.0- 6.0) | 5.0 (4.0-6.0) | 5.0 (4.0- 6.0) | 5.0 (4.0-6.0) | 4.0 (4.0-6.0) |
| 1-3 cycles, n (%) | 67 (17.0) | 35 (18.9) | 19 (15.6) | 11 (16.9) | 2 (9.1) | 44 (21.1) |
| 4 cycles, n (%) | 114 (28.9) | 64 (34.6) | 28 (23.0) | 16 (24.6) | 6 (27.3) | 81 (38.8) |
| 5-6 cycles, n (%) | 133 (33.8) | 45 (24.3) | 53 (43.4) | 23 (35.4) | 12 (54.5) | 48 (23.0) |
| ≥7 cycles, n (%) | 80 (20.3) | 41 (22.2) | 22 (18.0) | 15 (23.1) | 2 (9.1) | 36 (17.2) |
| **Dose reduction, n (%)** | 86 (21.8) | 39 (21.1) | 30 (24.6) | 14 (21.5) | 3 (13.6) | 59 (28.2) |
| First cycle when dose reduction occurred, median (IRQ) | 3 (2-4) | 3 (2-4) | 3 (2-4) | 3 (2-5) | 4 (2-4) | 3 (2-4) |
| **Changed platinum agent, n (%)** | 35 (8.9) | 22 (11.9) | 7 (5.7) | 5 (7.7) | 1 (4.5) | 22 (10.5) |
| First cycle when platinum agent was changed, median (IRQ) | 4.0 (3.0- 5.0) | 4.5 (3.0-6.0) | 3.0 (2.0-4.0) | 4.0 (3.0-4.0) | 4.0 (4.0-4.0) | 3.0 (2.0-4.0) |
| **PBC dosing period, median (IQR), weeks^†^** | 22.1 (17.0-34.1) | 20.3 (16.0-30.0) | 23.1 (17.4- 31.1) | 31.3 (18.7-59.6) | 23.7 (20.0- 53.1) | 22.0 (16.4-30.3) |
| **Treatment-free interval, median (IQR), weeks^‡^** | 5.0 (3.1-6.7) | 4.4 (3.0-6.9) | 4.7 (3.9-6.0) | 5.6 (5.0-7.3) | 3.9 (3.0-5.4) | 5.0 (3.9-6.9) |
| <4 weeks, n (%) | 127 (32.2) | 71 (38.4) | 35 (28.7) | 9 (13.8) | 12 (54.5) | 59 (28.2) |
| 4-10 weeks, n (%) | 246 (62.4) | 108 (58.4) | 83 (68.0) | 47 (72.3) | 8 (36.4) | 131 (62.7) |
| >10 weeks, n (%) | 21 (5.3) | 6 (3.2) | 4 (3.3) | 9 (13.8) | 2 (9.1) | 19 (9.1) |
| **No. of avelumab cycles, median (IQR)** | 6.0 (4.0-11.0) | 7.0 (5.0-13.0) | 6.0 (4.0-8.0) | 6.0 (4.0-11.0) | 8.5 (5.0-14.0) | 7.0 (3.0-14.0) |
| ≤4 cycles, n (%) | 115 (29.2) | 46 (24.9) | 42 (34.4) | 22 (33.8) | 5 (22.7) | 70 (33.5) |
| 5 to ≤12 cycles,  n (%) | 200 (50.8) | 91 (49.2) | 69 (56.6) | 29 (44.6) | 11 (50.0) | 78 (37.3) |
| 13 to ≤26 cycles, n (%) | 62 (15.7) | 37 (20.0) | 9 (7.4) | 11 (16.9) | 5 (22.7) | 47 (22.5) |
| ≥27 cycles, n (%) | 17 (4.3) | 11 (5.9) | 2 (1.6) | 3 (4.6) | 1 (4.5) | 14 (6.7) |
| **Avelumab maintenance dosing period, median (IQR), weeks^§^** | 18.5 (12.3-32.7) | 22.1 (14.1-40.1) | 15.1 (11.1-24.1) | 16.4 (12.1- 31.3) | 20.6 (13.3-30.1) | - |
| ≤8 weeks, n (%) | 31 (7.9) | 11 (5.9) | 13 (10.7) | 7 (10.8) | 0 | - |
| >8 to ≤24 weeks, n (%) | 212 (53.8) | 86 (46.5) | 77 (63.1) | 36 (55.4) | 13 (59.1) | - |
| >24 to ≤52 weeks, n (%) | 115 (29.2) | 66 (35.7) | 26 (21.3) | 16 (24.6) | 7 (31.8) | - |
| >52 weeks, n (%) | 36 (9.1) | 22 (11.9) | 6 (4.9) | 6 (9.2) | 2 (9.1) | - |

*Treatment with a cisplatin- or carboplatin-containing regimen other than cisplatin + gemcitabine, carboplatin + gemcitabine, or ddMVAC.

^†^Time from the first dose of 1L PBC to the first dose of avelumab maintenance treatment.

^‡^Time from the last dose of 1L PBC to the first dose of avelumab maintenance treatment.

^§^Time from the first dose of avelumab to the first dose of 2L treatment.

**Table S3. Subsequent treatments after avelumab maintenance.** Abbreviations: 1L, first line; 2L, second line; 3L, third line; 4L, fourth line; 5L, fifth line; ddMVAC, dose-dense methotrexate, vinblastine, doxorubicin, and cisplatin; EV, enfortumab vedotin; PBC, platinum-based chemotherapy.

|  | **Patients who received 2L treatment (n=394)** | **Type of 2L treatment** | | | |
| --- | --- | --- | --- | --- | --- |
|  |  | **EV (n=185)** | **PBC (n=122)** | **Pembrolizumab (n=65)** | **Other (n=22)** |
| **Lines of treatment, n (%)** |  |  |  |  |  |
| Received 2L | 394 (100) | 185 (100) | 122 (100) | 65 (100) | 22 (100) |
| Received 3L | 144 (36.5) | 40 (21.6) | 72 (59.0) | 20 (30.8) | 12 (54.5) |
| Received 4L | 49 (12.4) | 9 (4.9) | 24 (19.7) | 7 (10.8) | 9 (40.9) |
| **1L PBC regimen, n (%)** |  |  |  |  |  |
| Cisplatin + gemcitabine | 236 (59.9) | 106 (57.3) | 79 (64.8) | 35 (53.8) | 16 (72.7) |
| Carboplatin + gemcitabine | 147 (37.3) | 71 (38.4) | 43 (35.2) | 28 (43.1) | 5 (22.7) |
| ddMVAC | 5 (1.3) | 4 (2.2) | 0 | 1 (1.5) | 0 |
| Others | 6 (1.5) | 4 (2.2) | 0 | 1 (1.5) | 1 (4.5) |
| **2L treatment, n (%)** |  |  |  |  |  |
| EV | 185 (47.0) | 185 (100) | 0 | 0 | 0 |
| Cisplatin + gemcitabine | 64 (16.2) | 0 | 64 (52.5) | 0 | 0 |
| Carboplatin + gemcitabine | 58 (14.7) | 0 | 58 (47.5) | 0 | 0 |
| ddMVAC | 0 | 0 | 0 | 0 | 0 |
| Pembrolizumab | 65 (16.5) | 0 | 0 | 65 (100) | 0 |
| Other | 22 (5.6) | 0 | 0 | 0 | 22 (100) |
| **Did not receive 3L treatment, n (%)** | 250 (63.5) | 145 (78.4) | 50 (41.0) | 45 (69.2) | 10 (45.5) |
| No further treatment after 2L | 145 (36.8) | 75 (40.5) | 32 (26.2) | 30 (46.2) | 8 (36.4) |
| Ongoing 2L treatment | 105 (26.6) | 70 (37.8) | 18 (14.8) | 15 (23.1) | 2 (9.1) |
| **3L treatment, n (%)** |  |  |  |  |  |
| EV | 49 (34.0) | 1 (2.5) | 28 (38.9) | 15 (75.0) | 5 (41.7) |
| Cisplatin + gemcitabine | 14 (9.7) | 11 (27.5) | 1 (1.4) | 2 (10.0) | 0 |
| Carboplatin + gemcitabine | 9 (6.3) | 7 (17.5) | 0 | 2 (10.0) | 0 |
| Pembrolizumab | 65 (45.1) | 19 (47.5) | 42 (58.3) | 0 | 4 (33.3) |
| Others | 7 (4.9) | 2 (5.0) | 1 (1.4) | 1 (5.0) | 3 (25.0) |
| **Did not reach 4L treatment, n (%)** | 95 (66.0) | 31 (77.5) | 48 (66.7) | 13 (65.0) | 3 (25.0) |
| No further treatment after 3L | 58 (40.3) | 19 (47.5) | 31 (43.1) | 6 (30.0) | 2 (16.7) |
| Ongoing 3L treatment | 37 (25.7) | 12 (30.0) | 17 (23.6) | 7 (35.0) | 1 (8.3) |
| **4L treatment, n (%)** |  |  |  |  |  |
| EV | 20 (40.8) | 0 | 16 (66.7) | 2 (28.6) | 2 (22.2) |
| Cisplatin + gemcitabine | 4 (8.2) | 1 (11.1) | 1 (4.2) | 1 (14.3) | 1 (11.1) |
| Carboplatin + gemcitabine | 3 (6.1) | 1 (11.1) | 0 | 1 (14.3) | 1 (11.1) |
| Pembrolizumab | 12 (24.5) | 3 (33.3) | 5 (20.8) | 2 (28.6) | 2 (22.2) |
| Others | 10 (20.4) | 4 (44.4) | 2 (8.3) | 1 (14.3) | 3 (33.3) |
| **Did not reach 5L treatment, n (%)** | 37 (75.5) | 7 (77.8) | 22 (91.7) | 4 (57.1) | 4 (44.4) |
| No further treatment after 4L | 26 (53.1) | 2 (22.2) | 18 (75.0) | 2 (28.6) | 4 (44.4) |
| Ongoing 4L treatment | 11 (22.4) | 5 (55.6) | 4 (16.7) | 2 (28.6) | 0 |

**Figure S1. Distribution of avelumab dosing intervals**

**
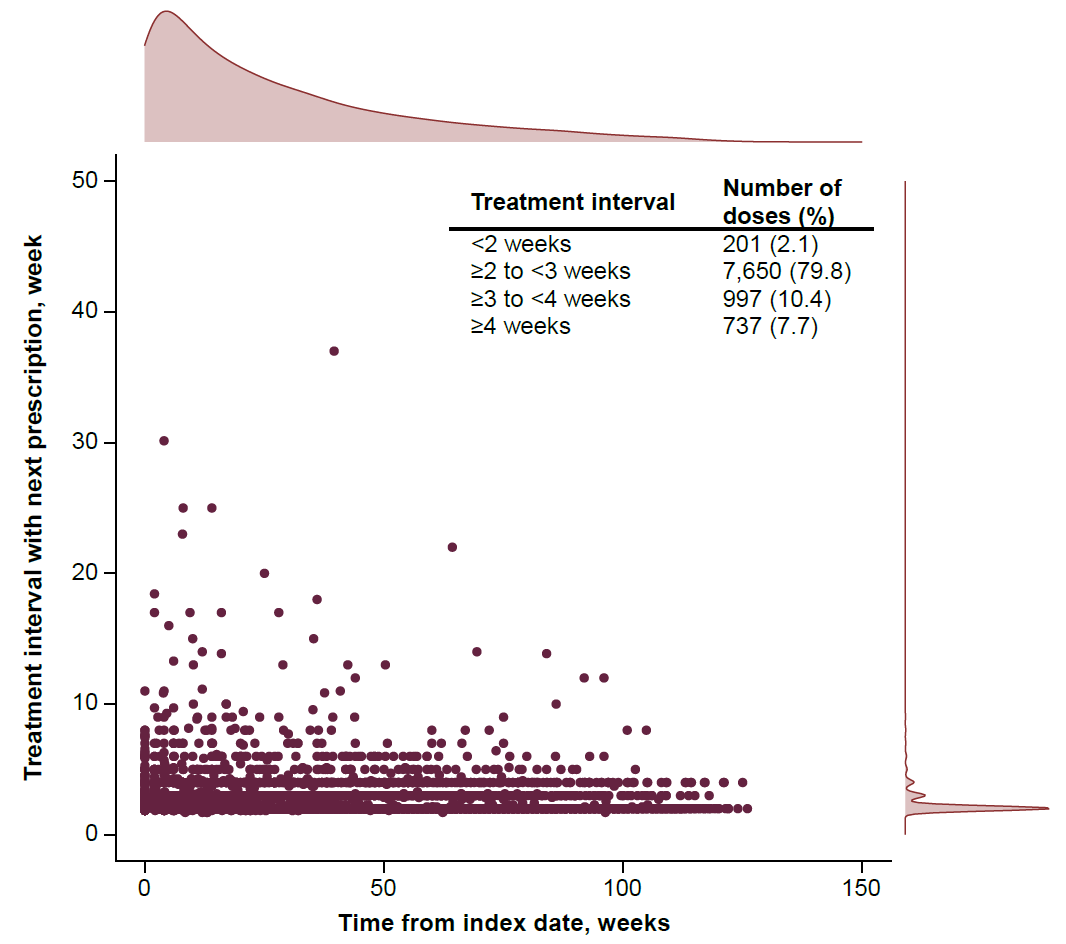
**Each dot represents the administration date of avelumab (horizontal axis) and the interval between an avelumab dose and the next avelumab dose (vertical axis).

The distribution of dosing intervals of avelumab maintenance therapy is shown on the right side, and the distribution of the administration date from the index date is shown on the upper side.

**Figure S2. Forest plot of TTF from the start of avelumab maintenance therapy in subgroups.** Abbreviations: 1L, first line; BMI, body mass index; ddMVAC, dose-dense methotrexate, vinblastine, doxorubicin, cisplatin; NE, not estimable; PBC, platinum-based chemotherapy; TFI, treatment-free interval; TTF, time to treatment failure.

**
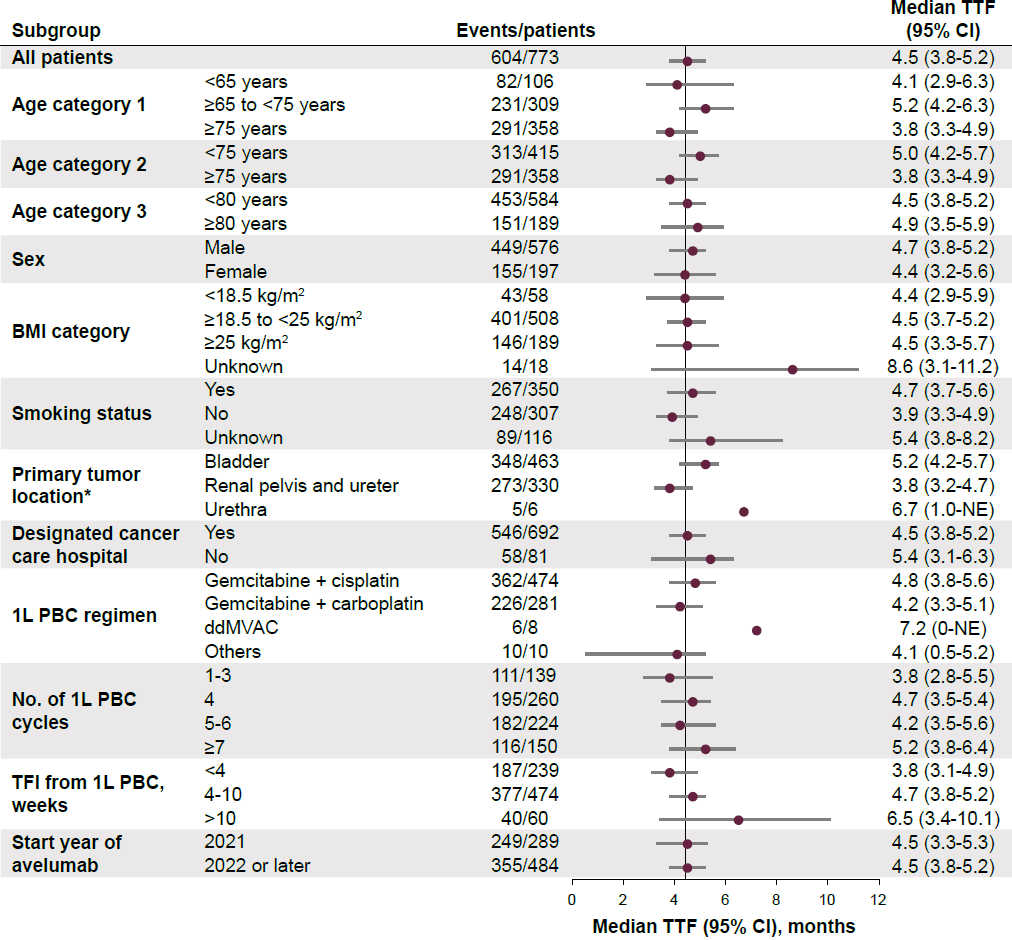
**
